# New Appliances and Things Medical

**Published:** 1899-02-11

**Authors:** 


### NEW APPLIANCES AND THINGS MEDICAL.

[We shall be glad to receive, at our Office, 28 & 29, Southampton Street, Strand, London, W.C., from the manufacturers, specimens of all new preparations and appliances which may be brought out from time to time.]

### IMPROVED MEDICAL BATTERY AND ACCESSORIES,

(NIBLETT AND SUTHERLAND, 31, CHANDOS STREET, STRAND, W.C.)

WE lately witnessed an interesting demonstration of the action of certain new electrical inventions which have a particular value from the point of view of the physician or surgeon, whose armamentarium has to include a means of working an electric cautery, or of effecting the electric illumination of larynx, eye, or internal cavity. The secondary battery is composed of cells which are practically of solid construction, consisting of earthenware plates, which are beset with ridges, those of two adjoining plates being at right angles, and thus containing between them a large series of minute chambers, which the electrolyte freely traverses in circulation, and which allows the ready escape of any gases generated during the charging or recharging of the cells. The conductor is of plain sheet lead, the battery can consist of any number of cells, and any current capacity engendered, but for medical purposes one of six cells with a capacity of 25 ampère hours will be found best adapted. In connection with this battery there is supplied an ingenious charging board, which clearly shows the direction of flow of the current—a precaution necessary unless the battery is to run the risk of destruction. Further than this, the charging board gives an approximate indication of the amount of current passing. Any form of battery can be charged from a direct current electric main, and the arrangement is such that any particular volt of charge is obtainable. These boards are applied for 50, 75, 100, 150, and 200 volts. A very useful form of resistance box is also supplied by the same firm; it is very finely graduated for variable resistance by means of a micrometric screw action. The value of such a device, which can reduce a pressure from 12 to 2 volts, and approximately in the same ratio with higher voltages, is obvious for those who have had any experience with the electric cautery or low voltage lamps, the merest shade of difference being of the utmost importance, a fineness of adjustment which can hardly be attained with any other resistance apparatus. The price of the whole apparatus is by no means excessive, and the secondary battery no more expensive than the usual variety, and decidedly more handy for travelling purposes.

### CASE PAPER. Designed by DR. J. K. COUCH and DR. E. LE C. LANCASTER.

(JOHN WRIGHT AND CO., BRISTOL.)

THESE case papers have been designed to facilitate the orderly record of notes taken at the bedside or in the consulting room. The space provided is not large, but there can be no doubt that the habit of filling up all the details for which room is provided will greatly tend to encourage a habit of methodical investigation. The special feature of these papers lies, if one may say so, overleaf, the back of each paper having printed on it a series of outline drawings of various parts of the body for the graphic record of physical signs. The papers are supplied at 15s. per 500, or bound in books of 250 copies, numbered and with index, at 11s.

